# Avian Influenza: Lessons from Past Outbreaks and an Inventory of Data Sources, Mathematical and AI Models, and Early Warning Systems for Forecasting and Hotspot Detection to Tackle Ongoing Outbreaks

**DOI:** 10.3390/healthcare12191959

**Published:** 2024-10-01

**Authors:** Emmanuel Musa, Zahra Movahhedi Nia, Nicola Luigi Bragazzi, Doris Leung, Nelson Lee, Jude Dzevela Kong

**Affiliations:** 1 Global South Artificial Intelligence for Pandemic and Epidemic Preparedness and Response Network (AI4PEP), Toronto, ON M3J 1P3, Canada; 2 Dahdaleh Institute for Global Health Research, York University, Toronto, ON M3J 1P3, Canada; 3 Africa-Canada Artificial Intelligence and Data Innovation Consortium (ACADIC), Toronto, ON M3J 1P3, Canada; 4 Department of Mathematics, York University, Toronto, ON M3J 1P3, Canada; 5 Department of Food and Drugs, University of Parma, 43125 Parma, Italy; 6 Canada Animal Health Surveillance System (CAHSS), Animal Health Canada, Elora, ON N0B 1S0, Canada; 7 Institute for Pandemics, Dalla Lana School of Public Health (DLSPH), University of Toronto, Toronto, ON M5S 1A1, Canada; nelsonls.lee@utoronto.ca; 8 Artificial Intelligence and Mathematical Modeling Lab (AIMMlab), DLSPH, University of Toronto, Toronto, ON M5S 1A1, Canada; 9 Institute of Health Policy, Management and Evaluation (IHPME), University of Toronto, Toronto, ON M5S 1A1, Canada

**Keywords:** avian influenza, H5N1, hotspot, mathematical modeling, artificial intelligence, conventional and unconventional data, early warning system

## Abstract

Background/Objectives: The ongoing avian influenza (H5N1) outbreak, one of the most widespread and persistent in recent history, has significantly impacted public health and the poultry and dairy cattle industries. This review covers lessons from past outbreaks, risk factors for transmission, molecular epidemiology, clinical features, surveillance strategies, and socioeconomic impacts. Since 1997, H5N1 has infected over 900 individuals globally, with a fatality rate exceeding 50%. Key factors influencing infection rates include demographic, socioeconomic, environmental, and ecological variables. The virus’s potential for sustained human-to-human transmission remains a concern. The current outbreak, marked by new viral clades, has complicated containment efforts. Methods: This review discusses how to integrate technological advances, such as mathematical modeling and artificial intelligence (AI), to improve forecasting, hotspot detection, and early warning systems. Results: We provide inventories of data sources, covering both conventional and unconventional data streams, as well as those of mathematical and AI models, which can be vital for comprehensive surveillance and outbreak responses. Conclusion: In conclusion, integrating AI, mathematical models, and technological innovations into a One-Health approach is essential for improving surveillance, forecasting, and response strategies to mitigate the impacts of the ongoing avian influenza outbreak. Strengthening international collaboration and biosecurity measures will be pivotal in controlling future outbreaks and protecting both human and animal populations from this evolving global threat.

## 1. Introduction

The avian influenza A (H5N1) virus infection (‘bird flu’) was first transmitted to humans in 1997 in Hong Kong, where 18 cases and 6 deaths were reported [1]. Since then, human cases have been reported to the World Health Organization (WHO), primarily from 15 countries in Asia, Africa, the Pacific, Europe, and the Middle East, though over 60 countries have been affected [1]. Human infections have remained relatively rare, but the virus has demonstrated its potential to move from one species to another, raising concerns that sustained human-to-human transmission could become possible [1,2]. Currently, human cases are occasionally detected in situations where avian influenza viruses are circulating in poultry. From 1 January 2003 to 3 May 2024, 889 cases of human infection with the avian influenza A (H5N1) virus were reported from 23 countries globally. Of these 889 cases, 463 were fatal, giving a case fatality rate of 52% [2,3].

Outbreaks of avian influenza A (H5N1) have been occurring in recent years, and the virus has been spreading in bird populations from Asia to Europe and Africa, and to the Americas in 2021, becoming endemic in poultry populations in many countries. Migratory wild birds, particularly the waterfowl, serve as a natural reservoir for avian influenza viruses, and they are able to contract the disease themselves and facilitate the transmission of the viruses across vast geographical areas [1]. The ongoing outbreaks are particularly concerning because various mammalian species are being infected, including seals, foxes, bears, cows, and even domestic cats. Furthermore, the avian influenza virus has multiple subtypes whose genetic characteristics are rapidly evolving. Since 2020, a new H5N1 strain has emerged and spread across continents (clade 2.3.4.4b) in 26 countries and acquired several mutations (*PB2* gene) adapted to infect up to 48 mammal species [3].

Human infections have primarily occurred through close contact with infected birds or contaminated environments, highlighting the importance of proper biosecurity measures and personal protective equipment when handling poultry or other potentially infected animals [1,2,3]. In the United States, since 2022, four human cases of avian influenza A (H5N1) were reported as of 26 June 2024, three of which were linked to exposure to dairy cows and one to infected poultry [2]. While these cases were mild, they serve as a stark reminder that the virus is constantly evolving and adapting, potentially gaining the ability to transmit more efficiently among humans.

As the world contends with the ongoing avian influenza A (H5N1) outbreak, this review aims to highlight lessons from past outbreaks in humans and animals, and identify and discuss current technological innovations and tools for forecasting and hotspot detection. Specifically, this review provides crucial insights on the lessons learned from past avian influenza outbreaks, identify conventional and unconventional data sources, and understand how diverse data sources, predictive models, and modern tools like artificial intelligence and machine learning could be leveraged for forecasting, hotspot detection, and early warning systems. These insights are essential for devising response strategies, especially in the context of ongoing outbreaks. They are also imperative for improving outbreak and pandemic preparedness, and the mitigation of the potential consequences of a future influenza pandemic, informing policymakers, and protecting both animal and human populations from devastating effects.

## 2. Lessons Learned from Past Avian Influenza Outbreaks

Valuable lessons have been learned from past avian influenza epidemics in various countries globally, but there are still knowledge gaps. One important lesson learned is that the avian influenza virus is zoonotic, meaning it can be transmitted from birds to humans. Infection with avian influenza viruses, such as the H5N1 and H7N9 strains, is particularly notable for causing severe disease in humans [4]. However, person–to–person transmission is uncommon, but when it occurs it can result in severe disease and death. Many of the signs and symptoms associated with avian influenza in humans have been identified and have proven invaluable in diagnosing and treating human H5N1 infection.

It has also been recognized that strict biosecurity implementation helps considerably reduce avian influenza viral transmission. This has been demonstrated during the control of avian influenza outbreaks within backyard flocks and commercial poultry farms [5,6,7]. Good hygiene, sanitary conditions, the disinfection of premises or culling chickens once infected, and proper handling practices can prevent spread [8].

The potential for influenza viruses to undergo genetic alteration by viral antigenic drift and shift is of concern, leading to a new strain that may be more virulent or escape existing immunity. Nonetheless, as was demonstrated in previous outbreaks, resilience is dependent on the strength of surveillance systems for early detection and response. To address the challenges of viral mutation and surveillance, the WHO established global systems for surveillance and response to increase the capacity to forecast, detect, and monitor influenza virus spread among humans—particularly avian flu viruses such as H5N1. The information gathered is also essential for expanding vaccine production. These developments have been vital in dealing with avian influenza outbreaks until now, and studies on human vaccines are still contributing to more preparedness for potential pandemics [9]. As this viral threat keeps changing, continuous research and surveillance are needed to stay one step ahead in preparedness.

## 3. Current Avian Influenza Outbreak

The ongoing avian influenza outbreak has been one of the most widespread and persistent in recent history because of its panzootic nature (large-scale infections among animals across continents), resulting in economic and biodiversity losses in a manner different from the situation in 2003–2019 [10,11]. Since its emergence in 2020, the virus has rapidly spread across multiple continents, affecting poultry populations, and causing significant economic losses in agriculture [11]. As of early 2024, it has spread to more than sixty countries in Africa, Asia, and Europe as well as the Americas with millions of domestic and wild birds being culled or dying from infection [10]. Successive waves of the outbreak have been characterized by novel viral clades and subclades differing to variable extents in pathogenicity and transmissibility. The first wave, which was fueled by the 2.3.4.4b clade, led to massive outbreaks in poultry farms and homesteads across Asia and Europe [12,13]. The emergence of additional subclades, followed by prolonged expansion and disease transmission, has made it even more difficult to control this outbreak [13]. 

### 3.1. Risk Factors for Avian Influenza Transmission

The avian influenza virus is largely spread among birds, and from birds or their secretions to humans directly [2,12]. Although the total number of confirmed human cases is by far very low compared to widespread avian exposure, a greater potential for emergence at some distant time always exists. Exposure to the avian influenza virus is almost always a major risk factor for animal and human health, due mainly to direct or indirect contact with live birds on farms, backyard poultry, or in markets. Direct exposure to infected birds (e.g., during the preparation of poultry for consumption) greatly increases human disease transmission [14,15]. Cooking thoroughly inactivates the virus; however, there is a low risk of spread from the consumption of raw or undercooked infected poultry products (including eggs), and unpasteurized milk from infected dairy. Virus transmission can occur through activities such as handling backyard poultry, visiting live poultry markets, or coming into contact with environments contaminated by the secretions or feces of infected birds, such as farms or animal markets. Additionally, shared domestic spaces, especially those with close physical interaction among individuals—such as households where someone is infected—can increase the risk of spreading the virus [2,14,15,16].

Some groups of people are at greater risk for exposure, among them poultry workers, veterinarians, and cullers who perform slaughter on infected birds or prepare sick animals [14,15]. Humans with certain underlying medical conditions such as chronic pulmonary diseases and those who have renal dysfunction or immunodeficiencies are known to be at increased risk of severe illness if they become infected [17].

### 3.2. Molecular Epidemiology and Viral Evolution

The H5N1 virus is a subtype of the influenza A virus, known for its ability to undergo genetic mutations and reassortment, leading to the emergence of new strains [18]. The ongoing outbreak has been characterized by the rapid evolution of the virus, with multiple clades and subclades emerging and co-circulating simultaneously [19]. Genetic analysis of the circulating strains has revealed mutations that may enhance the virus’s ability to infect and replicate in mammalian hosts, raising concerns about its potential for increased human transmissibility [20]. Continued monitoring and surveillance of the virus’s genetic changes are crucial for understanding its evolutionary trajectory and informing prevention and control strategies [13,18].

### 3.3. Clinical Features of H5N1 Infection

Avian influenza virus (H5N1) infection in humans leads to respiratory symptoms including fever, cough, and breathing difficulty. While the virus is usually associated with mild symptoms, these can progress to severe complications such as pneumonia, acute respiratory distress syndrome (ARDS), severe hypoxemia, and multiple organ failure syndrome (MOFS) [19,21]. Some concerns about missing asymptomatic or mildly symptomatic cases, giving a lower infection fatality ratio (IFR), have been raised [22,23,24], but available evidence suggests that these may be few. 

The case fatality rate of H5N1 infections in humans has been alarmingly high with a range of 50–60% [19]. The severity of illness results from the capacity of the virus to evoke a vigorous inflammatory response (a ‘cytokine storm’) and an ability for opportunistic secondary bacterial infections [23].

### 3.4. Surveillance Strategies and Public Health Control Measures

Surveillance and detection strategies are key in tracking the spread of avian influenza to identify new cases rapidly. These systems provide data on zoonotic virus circulation. While animal surveillance systems can monitor live markets and wild bird populations, as well as poultry farms, human health surveillance systems are based on case reporting, syndromic surveillance, and laboratory testing [17,25,26]. Both surveillance systems play a vital role in early detection and rapid outbreak containment but under-reporting and limited access to diagnosis and healthcare could impede these efforts [25,26].

Public health interventions and control measures to stop avian influenza spread require multifaceted approaches. Biosecurity is crucial in poultry and live animal markets to prevent transmission [27,28]. High-risk groups such as poultry workers or those who come in contact with sick birds/animals must wear personal protective equipment (PPE) and strictly comply with infection prevention and control measures [15]. Antivirals (e.g., oseltamivir) can be used for both treatment and prevention in some circumstances [29,30,31,32]. Vaccine development is ongoing, with many candidate vaccines currently in various stages of clinical trials [33]. However, the rapid evolution of the virus and frequent changes to respond to circulating strains present a significant challenge for producing and deploying a vaccine [34].

## 4. Mathematical Modeling of Avian Influenza

Mathematical models are crucial for understanding and controlling infectious diseases like avian influenza, providing quantitative frameworks for simulating disease spread and evaluating control strategies [33]. A recent review by Kirkeby and Ward highlighted the use of mathematical modeling to predict the spread of avian influenza viruses within and between poultry flocks [35]. The review found significant variability in transmission parameters, such as the basic reproduction number, latent period, and infectious period, influenced by factors like virus type, pathogenicity, species, study type, and poultry flock unit. The basic reproduction number varied widely across studies, with the highest estimates for H5N1 and H7N3 viruses. It was higher for within-flock transmission compared to between-flock transmission and higher for ducks compared to other species. Field studies generally reported higher values than experimental studies. The median latency period was around 1 day, while the infectious period ranged from 6.35 days for within-flock transmission to 9.6 days for between-flock transmission. 

Accurate parameterization is essential for reliable simulation models, requiring continuous updates and validation through ongoing research. Among the various types of models, mathematical or analytical models, such as the “Susceptible–Exposed–Infectious–Recovered” (SEIR) model, provide a quantitative framework for understanding avian influenza transmission dynamics. These models, based on data from real epidemics or experiments, are useful for describing epidemics and understanding pathogen behavior within populations. Another type of modeling is represented by spatial models, which can account for the geographical distribution of farms and disease spread dynamics, providing more accurate details on virus introduction estimation and contact tracing in a given geographical area, illustrating how spatial factors influence transmission rates and control measures’ effectiveness. 

Within-host models focus on virus dynamics within individual birds, integrating genomics data—such as transcriptomic, proteomic, and metabolomic data—across different biological scales and timeframes. Systems biology aims to build predictive models of H5N1 infection by examining interactions at multiple levels, from molecular to organismal. This involves high-throughput technologies to generate large datasets analyzed through computational models, uncovering emergent properties not predictable by studying individual components in isolation. These models help understand the host’s transcriptomic response at the cellular or lung tissue level during H5N1 infection, revealing critical aspects of the innate immune response and immune cell infiltration [36]. 

Predictive models simulate the effects of various control measures on epidemic courses, assisting policymakers in implementing effective measures. However, they rely heavily on real data, which are often insufficient, necessitating additional experiments or outbreak data analyses. These models, more complex than analytical models, can incorporate expert opinions when data are lacking [37]. 

Key insights from published mathematical models show that enhanced surveillance is crucial in controlling avian influenza outbreaks. These measures, combined with the depopulation of infected and surrounding flocks, significantly reduce disease spread. The immediate depopulation of infected flocks is consistently identified as the most effective control strategy, and strategic vaccination can effectively halt epidemic progression. Estimating the time and source of virus introduction is vital for efficient control measures. Accurate contact tracing and farm-specific interventions can drastically reduce epidemic size and duration. Implementing seasonal sampling and preventive measures during high-risk periods can improve the detection and control of avian influenza. 

Combining multiple control strategies, such as increased surveillance and immediate depopulation, coupled with vaccination strategies, leads to significant reductions in outbreak duration and infected flocks. Early detection and rapid response are critical for minimizing avian influenza spread. Predictive modeling assists in preparing and implementing effective measures. Ongoing field studies and controlled experiments are necessary to refine models and validate assumptions, ensuring effective control strategies under real-world conditions.

## 5. Machine Learning Models for Avian Influenza

Machine learning models have extensively been deployed for modeling, analyzing, and controlling avian influenza (Table 1). A wide group of these methods focus on finding demographic, socioeconomic, and environmental factors that are associated with infection or mortality of bird flu [38,39,40,41,42,43,44,45,46,47,48]. Among these risk factors, lower temperature, humidity, higher farm density, poultry density, bird density, and human population are commonly reported as the most important factors associated with a high number of cases [49,50,51,52,53]. Kilpatrick et al. [54] used regression analysis to discover whether H5N1 infections in different countries are the result of poultry or migratory birds. They found that, unlike Asia where H5N1 was mainly caused by poultry, the virus had spread in Europe through migratory birds, while in Africa the infection was partly caused by poultry and partly by migratory birds. Some other works have studied the environmental factors that ease the transmission of avian influenza from birds to other species such as dogs, cats, and pigs [55,56].

Machine learning techniques are also used to assess the effectiveness of interventions for reducing avian flu infection and mortality [57,58,59,60,62,98], including vaccines [61,63,64]. Machine learning techniques are also applied to explore the genomic properties of the avian influenza virus, its sub-variants, and mutations [39,65,66,67,68,69,72,73]. For instance, Chadha et al. [70] developed a Convolutional Neural Network (CNN) model to predict the pathogenicity of H5N1 virus for poultry species. Islam et al. [71] used multivariate logistic regression to detect the prevalence of the avian influenza virus in various waterfowls. 

Moreover, machine learning techniques have been used to study avian influenza infection on both spatial [41,80,81,83,84,99] and temporal dimensions [74,75,76,77,85]. Unconventional sources of data such as Google Trends, number of news articles, and number of social media posts are often found to be significantly correlated with the number of disease infections or fatalities. Therefore, recent studies have employed unconventional web-based data to forecast outbreaks and infection peaks. For example, Lu et al. [78] used multiple linear regression on a temporal level to build an early warning system for avian influenza outbreaks based on Google Trends. 

On a spatial level, a large volume of the literature concentrates on identifying environmental factors that make a particular geo-location suitable for migratory bird survival [41,81,83,84]. These hotspots potentially increase the risk of avian influenza transmission from wild birds to domestic waterfowl or other species. Therefore, there is a higher risk of avian influenza at farms and poultries that are located in wild waterfowl habitats. Among a variety of different ecological and environmental factors, population density, mountain ranges, proportion of river size, and air temperature are commonly identified as factors ensuring habitat suitability for migratory birds [86,87]. In this context, spatial regression analysis is particularly used for identifying environmental factors and hotspots of migratory bird habitats [82,99].

In addition, Ecological Niche Modeling (ENM) methods such as MaxEnt are also used for estimating factor importance and area suitability for migratory bird survival [88,89,91]. Belkhiria et al. [90] compared MaxEnt and Random Forest to identify the main hotspots of wild waterfowls. Finally, multiple works have studied the association between time and space during avian influenza outbreaks [92,93,94,95,96,97]. For example, Azat et al. [82] designed a permutation space–time model to find a wave-like steady spread of H5N1 infection from north to south over time in Chile. 

## 6. Data Inventory

For conducting explorations on H5N1 (avian influenza), various data sources and types of data inventories can be utilized. These sources cover surveillance and clinical reports (Table 2), genetic sequences and epidemiological data (Table 3), and poultry trades, food safety, and waterfowl abundance information (Table 4). Key sources and inventories to consider include institutional websites, such as the WHO, which provides reports and updates on influenza activity, including H5N1, through its Global Influenza Programme and FluNet, a global web-based tool for influenza virological surveillance. The Centers for Disease Control and Prevention (CDC) offers data and statistics on influenza viruses, including H5N1, through its Influenza Division and FluView, a weekly influenza surveillance report. The European Centre for Disease Prevention and Control (ECDC) monitors influenza activity and provides epidemiological updates through its Influenza Surveillance program. The Food and Agriculture Organization (FAO) provides data on animal disease outbreaks, including avian influenza, through its EMPRES-i (Emergency Prevention System for Transboundary Animal and Plant Pests and Diseases).

For genetic and genomic data, the Global Initiative on Sharing All Influenza Data (GISAID) is a platform for sharing genetic sequences of influenza viruses, including H5N1. The National Center for Biotechnology Information (NCBI) offers a comprehensive database of genetic sequences, including influenza A (H5N1), through GenBank and the Influenza Virus Resource, which provides tools for the analysis of influenza sequences. The Influenza Research Database (IRD) is a comprehensive database providing sequence data, annotated data sets, and tools for influenza research. For clinical and research data, PubMed is a database of scientific publications where studies related to H5N1 can be found. ClinicalTrials.gov is a database of clinical studies related to H5N1, including ongoing and completed trials. For surveillance and monitoring reports, ProMED-mail is an internet-based reporting system dedicated to rapid global dissemination of information on outbreaks of infectious diseases. HealthMap is a global disease alert map that provides real-time surveillance of emerging public health threats, including avian influenza. 

Regional and national data sources include the China Animal Health and Epidemiology Center (CAHEC), which provides data on animal health, including avian influenza outbreaks in China. The Japan Ministry of Agriculture, Forestry and Fisheries (MAFF) offers reports on avian influenza outbreaks and control measures in Japan. The Animal and Plant Health Inspection Service (APHIS) of the USDA provides surveillance reports and data on avian influenza in the United States. 

For data inventory and management, the FAIR Data Principles ensure that data is Findable, Accessible, Interoperable, and Reusable. Utilizing FAIR principles can help in managing H5N1 data inventories effectively. Data repositories such as Dryad and Figshare allow researchers to share data, including data related to H5N1 research. By leveraging these diverse sources and inventories, researchers can gain comprehensive insights into the epidemiology, genetics, clinical aspects, and surveillance of H5N1, aiding in the exploration and understanding of this virus.

Two sources of data play a pivotal role in studying avian influenza morbidity and mortality, data gathered for animal infection surveillance, and clinical data gathered from human avian influenza infections [100]. Mostly, predictive models are developed using data collected from sick or dead birds or mammalians. Nevertheless, samples taken from domestic and peri-domestic environments such as faeces, mud, soil, water, feathers, and air, or poultry instruments such as cages, feeding sources, chopping boards, and de-feathering machines are mostly suitable for studying disease circulation, transmission routes, intervention effectiveness, risk assessment, and performing molecular analyses, such as gene analysis [101].

Clinical data which may include the number of cases, deaths, patient demographics, health history, socioeconomic factors, genetic and genomic sequences, and antibodies found in people could also be used to build predictive models for infection and fatality rates, detecting relevant predictors, and studying intervention and preventive policy effectiveness [102]. These sources of data are frequently combined with ecological, and environmental data taken from sources such as satellite information and weather or air quality sensors to analyze avian influenza infections on a spatial-temporal level, detecting hotspots and possible spill-overs, and extracting potential predictors [56,71,74,75,76,77,80,81,83,84,85,99]. Inter- and intra-country poultry networks are also another data source that is commonly combined with other sources of data for building surveillance, risk assessment, and predictive models [54,103,104,105]. 

Besides such conventional data, unconventional sources, such as social media posts, news articles, emails, and other web-based sources have also been considered for analyzing bird flu [78,106,107]. Several works have used tweets to forecast infection trends or mine mass opinions, detect fake news, mis- and dis-information regarding avian influenza [108,109]. News articles have also been gathered and analyzed for building predictive models, risk management, and preparedness [79,107]. 

## 7. Socioeconomic and Environmental Impacts

The ongoing avian influenza outbreak could lead to major socioeconomic and environmental impacts. Some control measures such as the culling of infected birds, trade restrictions, and decreased consumer demand have resulted in considerable economic losses in the poultry industry. Livelihoods and food security are severely impacted, particularly in rural areas where commercial or simple backyard-based poultry farming is an important source of income and dietary protein. Concerns about the environmental consequences, including the disposal of culled birds and wild bird populations being disrupted by raptor species are other potential risks [110,111]. Consequently, interventions in these aspects to enhance the resilience of the poultry industry are crucial in mitigating such impacts and promoting sustainable practices [27].

**Figure 1:**
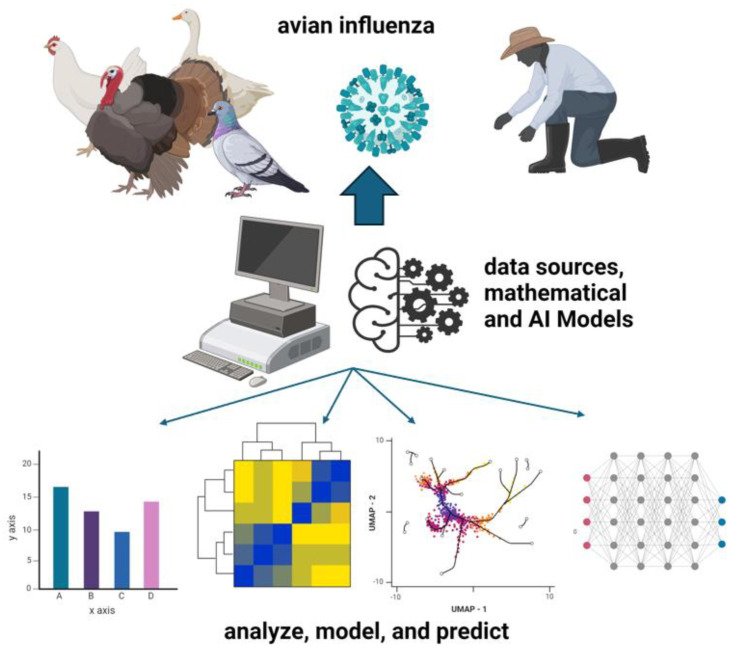
AI and mathematical models in analyzing, modeling, and predicting avian influenza transmission from diverse data sources, integrating biological insights from affected bird species and human interaction. This figure was created with BioRender.com.

Artificial intelligence, machine learning, and advanced mathematical models can significantly contribute to addressing the socioeconomic and environmental impacts of outbreaks such as avian influenza. As previously mentioned, these technologies can enhance the accuracy and speed of epidemiological modeling, allowing for the tracking and prediction of outbreaks based on large-scale datasets (Figure 1). By analyzing factors like weather conditions, bird migration patterns, and poultry movement, modeling can identify high-risk zones and provide early warnings, reducing the risk of outbreaks escalating into larger crises.

In addition, artificial intelligence-driven models can optimize control measures by simulating various interventions, such as selective culling, vaccination, or trade restrictions. These simulations allow for the evaluation of potential outcomes, ensuring that the most effective strategies are chosen while minimizing economic disruption and safeguarding livelihoods. Artificial intelligence can also enhance the efficiency of supply chains during an outbreak, helping reduce food security risks in rural areas dependent on poultry farming. By analyzing consumer demand and logistics, artificial intelligence-based systems can better manage resources and maintain market stability.

From an environmental perspective, artificial intelligence and machine learning can help monitor the impact of control measures, such as the culling of infected birds, by analyzing disposal methods and assessing risks to wildlife populations. By predicting and mitigating the ecological consequences of these actions, artificial intelligence contributes to more sustainable practices in managing outbreaks.

Ultimately, artificial intelligence and mathematical models offer powerful tools for making informed decisions, enabling proactive measures that enhance the resilience of the poultry industry and reduce the overall impact of disease outbreaks on society and the environment.

## 8. Pandemic Preparedness and Response

The ongoing avian influenza outbreak has highlighted the importance of pandemic preparedness and response planning [15,34]. Even though sustained human-to-human transmission has not been observed, the risk of the virus acquiring the ability to spread efficiently among humans remains a significant concern [20]. Consequently, forecasting, hotspot detection, and preparedness planning which involves developing comprehensive strategies for surveillance, risk assessment, healthcare system readiness, and coordination among various stakeholders must be strengthened using the One-Health approach [29,34]. International collaboration and information sharing are also crucial for effective pandemic response, as demonstrated by the efforts of organizations like the WHO and the FAO [112,113].

## 9. Challenges and Future Directions

An emerging issue in the current avian influenza panzootic outbreak that has caused unprecedented mass fatalities, is the transmission of avian influenza to cattle and mammals [114,115,116]. In this context, domestic animals such as cats and dogs are of especial concern, since they are in direct contact with humans and could easily transmit it [55,56]. It is paramount to study the genes and genomic sequences of this strain, perform risk analysis, and identify the amino-acid mutations that result in transmissibility to humans and other mammalians [66]. Although a great volume of the literature explores the pathogenicity and prevalence of avian influenza in different waterfowls [39,67,68,69,70,71,73,99], very few studies concentrate on mammals including humans [66,72].

Previously, regulations such as house ordering [58,59,60,62], hygienic and biosecurity practices in poultry and live bird markets [69], and mass poultry vaccination [61,63,64] have been implemented to reduce avian influenza cases in poultries and bird farms. Similar measures and policies need to be set and evaluated to prevent or mitigate avian influenza infections in domestic cattle and mammals [39].

In addition, biological and environmental factors that increase the risk of transmission of avian influenza to mammals need to be identified. A rich literature on risk factors associated with avian influenza is already available that elucidates variables such as ambient temperature [49,94], closeness to water [38,40,43,48,88,89,90,91], waterfowl abundance [42,51,52,94,95,96,97], human population [51], poultry density and sanitation [48,50], and farmers and poultry workers knowledge [41,44,46,47] as the most significant features. Such studies need to be extended to recognize the risk factors, hotspots, and spatial and temporal characteristics of mammalian infections. More datasets on cattle and mammal routes and pathways must be collected on a global level to improve the accuracy of the predictions.

Another concern in this field that very limited studies focus on [38,94] is how climate change affects the spatial and temporal trends of avian influenza. Climate change, urbanization, and deforestation are shifting the bird migration patterns and even regional movements. Species Distribution Modeling (SDM) and spatiotemporal analysis need to be continuously applied to track the deviations in avian influenza outbreak patterns.

Another area that has potential for further research is retrieving and analyzing posts from social media platforms such as Reddit, Facebook, and Twitter. Since such studies provide information on hot trends, mass opinions, concerns, and mis- and dis-information, they are of particular interest to policy-makers, health officials, social workers, and other parties. However, a very limited number of studies have focused on analyzing social media posts regarding avian influenza [106,107]. Web-based data such as social media posts, Google Trends [79], emails, page visits, and news articles are also a great resource for disease surveillance; yet, few papers have considered employing them for surveillance of avian influenza [79,107,108,109].

Another useful source of data that could help with avian influenza surveillance but has been neglected is data gathered from wastewater [117,118]. Recent studies have reported that using wastewater data, outbreaks could be detected by up to 17 days in advance [119].

Although the bulk of studies have focused on surveillance of avian influenza [21,53,100,101,120], limited number of works have tried to build early warning systems or forecasting models [54,74,75,76,77,78,79]. Moreover, methodologies need to be designed and implemented to improve the accuracy of existing models by eliminating false positives. Reducing the number of false positive avian influenza cases from predictive models is, indeed, very important, because it prevents unnecessary culling and poultry trade prohibitions [62].

Despite many advances in the knowledge and response to avian influenza outbreaks, several challenges still lie ahead. Improving the understanding of the virus’ evolutionary potential, transmission dynamics, and host–pathogen interactions through research, surveillance, and the application of modern tools like artificial intelligence, and machine learning is crucial. This will ensure that prompt detection and response to outbreaks can occur [57,58,59,60,62,98]. Enhancing surveillance systems for both animal and human populations [17,25,26,27], strengthening the biosecurity measures, and building local, national, and international level preparedness measures for pandemics are critical needs that would protect against further spread or resurgence of such outbreaks in the future [27,28].

## 10. Limitations

The limitations of this review include its narrative structure, which lacks the quantitative rigor typically found in systematic reviews or meta-analyses. A more quantitative approach, such as a meta-analysis, would allow for a more robust synthesis of data from multiple studies, offering stronger, statistically supported conclusions. Without this, the findings in this review may be subject to selective reporting and potential bias in the interpretation of individual studies.

Furthermore, the landscape of technological advancements, particularly in artificial intelligence tools and methodologies, is constantly evolving. This rapid development presents a challenge, as models and tools discussed in the review may become outdated quickly. As new algorithms, data sources, and artificial intelligence techniques emerge, the models and early warning systems for forecasting and hotspot detection must continually be updated to remain effective. The evolving nature of artificial intelligence technology may lead to gaps in this review’s applicability over time, requiring continuous reassessment to incorporate new tools and methods.

Additionally, the methodologies applied in different studies vary across temporal and spatial scales, leading to inconsistencies in findings. This makes it difficult to compare results and draw generalized conclusions across different outbreaks, regions, or time periods. This review also highlights the need for future studies to adopt a more systematic, data-driven approach, ensuring that insights are comprehensive and remain relevant as artificial intelligence and technological tools continue to advance.

## 11. Conclusions

In conclusion, leveraging advancements in technological tools, such as artificial intelligence and machine learning, offers significant potential in forecasting, hotspot detection, and early warning systems for managing avian influenza outbreaks. However, these tools must be integrated into a broader, collaborative effort that includes the One-Health approach, bringing together human, animal, and environmental health data. The ongoing avian influenza outbreak underscores the critical need for continuous technological innovation, international collaboration, and robust data-sharing among health authorities, veterinary services, environmental agencies, and research institutions. 

While artificial intelligence-driven models have shown promise in improving predictive accuracy and outbreak preparedness, they require ongoing refinement and adaptation to account for the rapidly evolving nature of viral threats and the dynamic challenges posed by environmental, socioeconomic, and biological factors. Furthermore, a concerted effort to bridge gaps in data quality, access, and collection will be crucial in making these technological advancements more reliable and universally applicable. Looking ahead, addressing the limitations of existing models through greater reliance on systematic, data-driven methodologies, and fostering stronger global collaboration will be vital in mitigating the socioeconomic and environmental impacts of future outbreaks. By combining these efforts, we can better safeguard public health, ensure food security, and promote more resilient agricultural practices in the face of increasingly complex pandemic threats [113,120,121,122].

## Figures and Tables

**Table 1:** Machine learning (ML) techniques for avian influenza. Abbreviations: Artificial Neural Networks (ANNs); Convolutional Neural Network (CNN); Genetic Algorithm for Rule-Set Prediction (GARP); Maximum Entropy (MaxEnt); Species Distribution Modeling (SDM); Single Shot MultiBox Detector (SSD); Support Vector Machine (SVM); Extreme Gradient Boosting (XGBoost); You Only Look Once (YOLO).

ML Method	Application	Animal Health	Human Health
Logistic regression [38,39,40,41,42,43,44,45,46,48,51,52], tobit regression [47], negative binomial regression [49], linear regression [52]	Identify animal and environmental risk factors associated with avian influenza occurrence	√	
Logistic regression [55,56]	Identify risk factors that result in the transmission of avian influenza from birds to mammalians such as dogs, cats, and pigs	√	
Logistic regression [45], Poisson regression [50], multivariable regression [53], linear regression [57]	Identify environmental, demographic, and socioeconomic risk factors associated with avian influenza occurrence		√
Linear regression [58], multilevel regression [59], birth process with regression model [60], logistic regression [61], SVM [62]	Study the efficiency of preventive policies such as poultry vaccination on the spread of the avian influenza virus among birds	√	
Cox proportional hazards regression [32], logistic regression [63,64]	Study the efficiency of pharmaceutical and non-pharmaceutical interventions on avian influenza transmission and mortality		√
Gradient boosted tree [65], SVM [66], multiple linear regression [67], simple regression [68], logistic regression [39,69,70,71]	Identify the molecular signatures that define the pathogenicity of viral strains	√	
Deep CNN [72], logistic regression [73]	Predict genomic sequences		√
Random Forest, Gradient Boosting, and XGBoost [74], SVM and ANN [75], binomial regression [76], and deep-learning models [77,78]	Predict avian influenza outbreaks in animals at the temporal level	√	
Multiple linear regression [79]	Forecast avian influenza outbreaks in humans at the temporal level		√
Bayesian logistic regression, XGBoost [41,80,81], spatial regression analysis [41,82], region-based CNN, SSD and YOLO [83], logistic regression [84,85], generalized linear mixed model [86], Poisson and logistic regression [87]	Identify geographical regions and risk factors of avian influenza hotspots	√	
MaxEnt [88,89,90], GARP [91], Random Forest [90]	Identify geographical and spatial factors of migratory bird hotspots and provide a risk map using SDM	√	
Linear regression and spatial regression [82], logistic regression [92,93,94,95], boosted regression tree [96], Poisson regression [97]	Analyze spatiotemporal factors affecting avian influenza	√	

**Table 2:** Dataset inventory for avian influenza surveillance.

Name of the Dataset	Author	Year Initiated	Description	Link
Global Influenza Programme (FluNet)	World Health Organization (WHO)	1997	Surveillance data on influenza including H5N1; from all over the globe; country-level; weekly basis	https://www.who.int/tools/flunet **(accessed on 30 September 2024)**
Emergency Prevention System for Transboundary Animal and Plant Pests and Disease (EMPRES-i)	Food and Agriculture Organization (FAO)	2004	Monitors wild and domestic animal disease including avian influenza; global-level; provides the exact coordinates of the incidence; daily basis	https://empres-i.apps.fao.org **(accessed on 30 September 2024)**
HPAI in Wildlife	Canadian Food Inspection Agency (CFIA)	December 2021	Number of avian influenza records in wild birds; Canada only; subdivision-level	https://cfia-ncr.maps.arcgis.com/apps/dashboards/89c779e98cdf492c899df23e1c38fdbc **(accessed on 30 September 2024)**
European Influenza Surveillance Network (EISN)	European Center for Disease Prevention and Control (ECDC)	2008	Surveillance data on influenza; In European Union (EU) and European Economic Area (EEA) countries; weekly basis	https://www.ecdc.europa.eu/en **(accessed on 30 September 2024)**
	World Organization for Animal Health (WOAH) (formerly known as the Office International des Epizooties (OIE))	1924	Provides data on zoonotic disease such as avian influenza; global level	https://www.woah.org/en/disease/avian-influenza/ **(accessed on 30 September 2024)**
	Government of United Kingdom	2022	Provides data on H5N1 and possibility of transmission from animals to humans or other mammalians; the UK	https://www.gov.uk/government/publications/avian-influenza-influenza-a-h5n1-technical-briefings **(accessed on 30 September 2024)**
	Ministry of Agriculture, Forestry and Fisheries (MAFF)	1881	Surveillance data on avian influenza outbreak; Japan	https://www.maff.go.jp/e/ **(accessed on 30 September 2024)**
	Center for Health Protection (CHP)	2004	Disease surveillance data including avian influenza surveillance data; Hong-Kong	https://www.chp.gov.hk/en/index.html **(accessed on 30 September 2024)**
Hospital Based Influenza Surveillance (HBIS)	Institute of Epidemiology, Disease Control and Research (IEDCR)	2012	Real-time influenza surveillance and hospitalization data; Bangladesh	https://www.iedcr.org/index.php?option=com_content&view=article&id=130&Itemid=86 **(accessed on 30 September 2024)**
National Influenza Surveillance in Bangladesh (NISB)	Institute of Epidemiology, Disease Control and Research (IEDCR)	2013	Real-time influenza surveillance data; Bangladesh	https://www.iedcr.org/index.php?option=com_content&view=article&id=131&Itemid=174 **(accessed on 30 September 2024)**

**Table 3:** Dataset inventory for genetic and genomic sequences.

Name of the Dataset	Author	Year Initiated	Description	Link
Global Initiative on Sharing Avian Influenza Data (GISAID)	World Health Organization (WHO) and other international and scientific organizations, e.g., the Association of Public Health Laboratories (APHL), the Swiss State Secretariat for Education, Research and Innovation (SERI), the Federal Office of Public Health (FOPH), and the U.S. Department of Agriculture (USDA)	2008	Includes viral genetic and genomic sequences and related epidemiological data of influenza viruses; global-level; country-level	https://gisaid.org **(accessed on 30 September 2024)**
Influenza Virus Resource	National Center for Biotechnology Information (NCBI)	2006	Provides data on avian influenza, including genomic sequences and related metadata; global-level; country-level	https://www.ncbi.nlm.nih.gov/labs/virus/vssi/#/virus?SeqType_s=Nucleotide&VirusLineage_ss=taxid:197911&VirusLineage_ss=taxid:197912&VirusLineage_ss=taxid:197913&VirusLineage_ss=taxid:1511083 **(accessed on 30 September 2024)**
Influenza Research Database (IRD)	National Institute of Health (NIH)/National Institute of Allergy and Infectious Disease (NIAID)	2008	Provides data on avian influenza including genomic sequences and related metadata; global-level; country-level	https://www.bv-brc.org/api/doc/ **(accessed on 30 September 2024)**
Avian Influenza DataBase (AIDB)	Supported by multiple organizations including World Health Organization (WHO), World Organisation for Animal Health (WOAH), and Food and Agriculture Organization of the United Nations (FAO)		Epidemiological, case report, and genomic data on avian influenza in real-time or near-real-time; global level; for regions with significant poultry industries and migratory bird populations	http://avian-flu.org **(accessed on 30 September 2024)**
FluGlobalNet	Supported by a coalition of international organizations, including World Health Organization (WHO) and Centers for Disease Control and Prevention (CDC)	2010	Provides epidemiological, genetic sequencing, vaccination coverage, and public health response data on avian influenza; global-level; country-level	https://science.vla.gov.uk/fluglobalnet/about_ai.html **(accessed on 30 September 2024)**
	China Animal Health and Epidemiology Center (CAHEC)	2002	Surveillance as well as genomic data on animal disease such as avian influenza; China only	https://www.cahec.cn **(accessed on 30 September 2024)**

**Table 4:** Dataset inventory for poultry trades, food safety, and waterfowl abundance.

Name of the Dataset	Author	Year Initiated	Description	Link
HPAI in Domestic Birds	Canadian Food Inspection Agency (CFIA)	2022	Number of avian influenza records in domestic birds in poultries and farms; Canada only; city-level	https://app.powerbi.com/view?r=eyJrIjoiMGZkNGRmZmQtNzg1My00ZmYxLTkzMTgtMWViNjg0MTBhYjRhIiwidCI6IjE4YjVhNWVkLTFkODYtNDFkMy05NGEwLWJjMjdkYWUzMmFiMiJ9 **(accessed on 30 September 2024)**
National Poultry Improvement Plan (NPIP) Database	NPIP and US Department of Agriculture’s (USDA) Animal and Plant Health Inspection Service (APHIS)	1935	Surveillance data on poultry disease including avian influenza, and international trade and export of poultry products from the USA; the USA	https://www.poultryimprovement.org/npipdatabase/Login/Npiplogin.cfm **(accessed on 30 September 2024)**
	European Commission in cooperation with the European Food Safety Authority (EFSA)	2002	Includes datasets on animal disease such as avian influenza, and risk associated with food chains; European Union countries and their member states	https://food.ec.europa.eu/animals/animal-diseases/diseases-and-control-measures/avian-influenza_en **(accessed on 30 September 2024)**
FAOSTAT	Food and Agriculture Organization (FAO)	1961	Provides data on trade statistics, food safety and supply, and animal disease such as avian influenza; country-level; annual basis	https://www.fao.org/faostat/en/#home **(accessed on 30 September 2024)**
PADI-web	Agency for Food, Environmental and Occupational Health & Safety (ANSES)	2015	A platform for animal disease such as avian influenza surveillance in wild and domestic birds in poultries and farms; France	https://www.padi-web-one-health.org **(accessed on 30 September 2024)**
Korean Animal Health Integrated System (KAHIS)	Korea’s Animal and Plant Quarantine Agency (APQA) and Ministry of Agriculture, Food and Rural Affairs (MAFRA)	2009	Surveillance data on animal disease such as avian influenza and trade, import, and export of poultry products to/from South Korea; South Korea	http://kahis.go.kr/ **(accessed on 30 September 2024)**
Danish Veterinary and Food Administration (DVFA)	Ministry of Environment and Food of Denmark	2011	Provides data on food safety and animal disease including avian influenza; Denmark	https://en.foedevarestyrelsen.dk **(accessed on 30 September 2024)**
	Ministry of Environment	2007	The number of migratory waterfowls; Japan	http://www.env.go.jp/nature/dobutsu/bird_flu/migratory/ap_wr_transit/index.html **(accessed on 30 September 2024)**
United Nations (UN) Comtrade	United Nations Statistics Division (UNSD)	1962	Provides annual trade data including poultry between countries, global-level; country-level	https://comtradeplus.un.org **(accessed on 30 September 2024)**
Poultry Industry Association of New Zealand (PIANZ)	The New Zealand poultry industry	1953	Provides data on poultry trade; New Zealand	https://www.pianz.org.nz **(accessed on 30 September 2024)**
Waterbirds Population Portal (WPP)	Wetlands International	2012	Provides data on population, distribution, habitat, and conservations status of waterbirds; for all countries where waterbirds are found	https://wpp.wetlands.org **(accessed on 30 September 2024)**
	Global Flyway Network (GFN)	2006	Provides data on migratory routes, population dynamics, breeding and wintering grounds, ecological studies, conservation status, and tracking data for different species of migratory birds; global flyways	https://www.globalflywaynetwork.org **(accessed on 30 September 2024)**
International trade data program	U.S. Census Bureau		Provides export and import data including poultry trades; the USA; monthly basis [54]	https://www.census.gov/foreign-trade/index.html **(accessed on 30 September 2024)**

## Data Availability

The data presented in this study are available at the URL/DOI and references in this manuscript. These data were derived from resources available in the public domains as shown in the list of references.
